# Six cases of refractory pruritus and histologic dermal hypersensitivity reaction successfully treated with dupilumab

**DOI:** 10.1016/j.jdcr.2021.10.030

**Published:** 2021-11-06

**Authors:** Nicole Edmonds, Margaret Noland, R. Hal Flowers

**Affiliations:** Department of Dermatology, University of Virginia, Charlottesville, Virginia

**Keywords:** chronic pruritus of unknown origin, dermal hypersensitivity reaction, dermatopathology, DHR, dupilumab, general dermatology, medical dermatology, CPUO, chronic pruritus of unknown origin, DHR, dermal hypersensitivity reaction

## Introduction

Dermal hypersensitivity reaction (DHR) is a histopathologic finding that characterizes many different clinical diagnoses, such as chronic pruritus of unknown origin (CPUO), drug reactions, arthropod bite reactions, spongiotic dermatitis, bullous pemphigoid, scabies, urticarial erythema multiforme, dermatitis herpetiformis, eosinophilic folliculitis, and urticarial vasculitis.^1^ Because DHR is a histologic rather than a clinical diagnosis, no single treatment regimen has been consistently used, and treatment has rather focused on addressing the underlying cause. CPUO is defined as an itch that lasts for greater than 6 weeks, that is not better explained by a dermatologic or other medical condition, and that can be associated with DHR and/or a spongiotic pattern on skin biopsy.^2^ Treatment of CPUO with DHR on histology is particularly challenging, as there is no primary disease to target. Topical therapy, oral antihistamines, systemic corticosteroids, antidepressants, and anticonvulsants have proven to be minimally effective for the treatment of CPUO.^3^ Dupilumab, an interleukin-4 receptor alpha antagonist approved for the treatment of atopic dermatitis, may be a viable treatment option for CPUO associated with DHR, given its effectiveness treating other T helper 2 cell-mediated diseases,^4^ such as idiopathic chronic eczematous eruption of aging, chronic prurigo, allergic contact dermatitis, hyper-eosinophilic syndrome, prurigo nodularis, and bullous pemphigoid. Here we present 6 cases of CPUO with biopsy findings consistent with DHR successfully treated with dupilumab, a potential novel treatment option for this challenging and poorly understood disease.

## Case series

### Case 1

**Fig 1:**
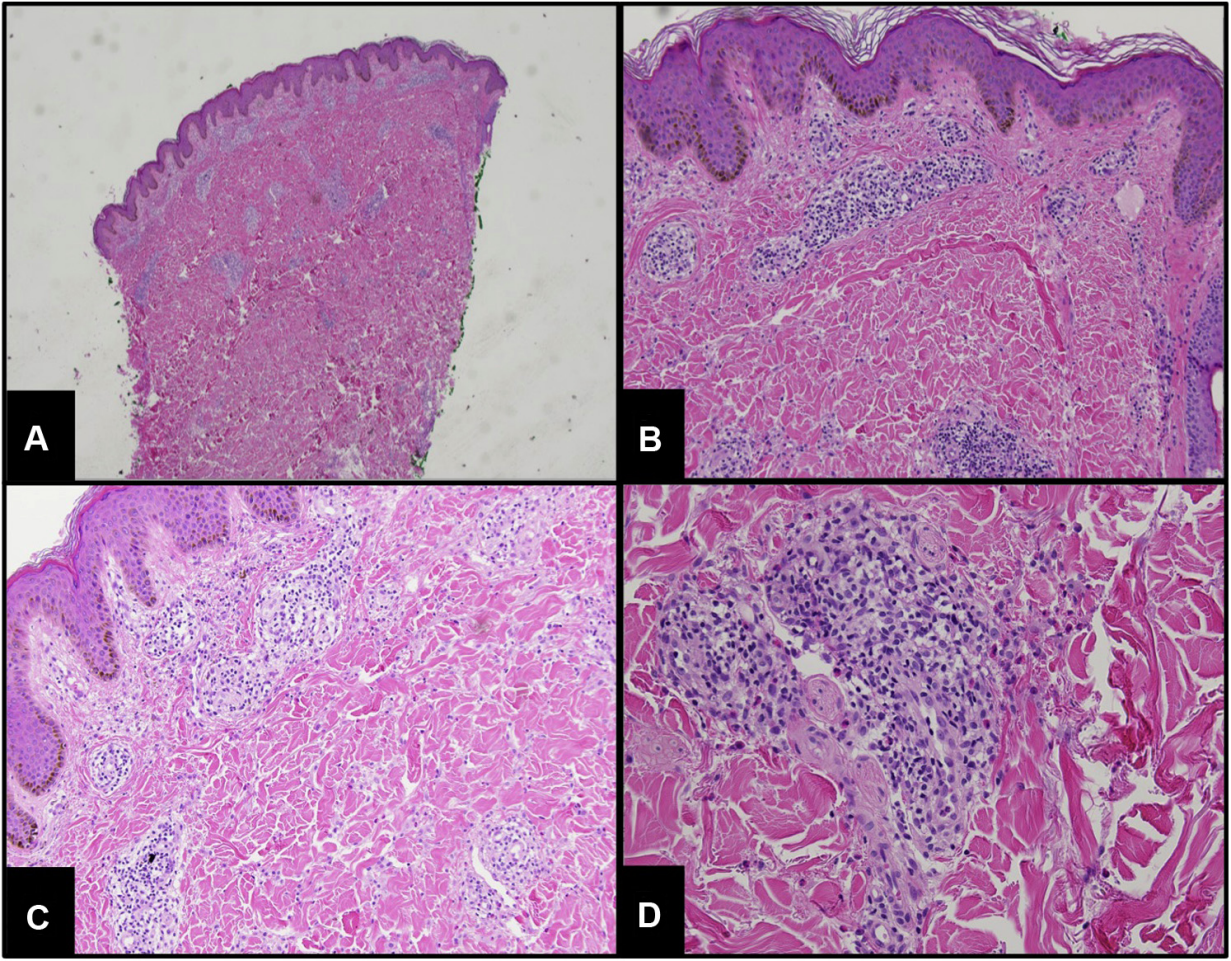
Punch biopsy of the left back of patient case 1. Demonstrations of a mildly spongiotic epidermis with lymphocytic inflammatory infiltrate with numerous eosinophils surrounding the vessels of the papillary dermis. (**A**, **B**, **C**, and **D**, Hematoxylin-eosin stain; original magnifications: **A**, ×2; **B**, ×10; **C**, ×10; and **D**, ×20.)

A 53-year-old man presented with a pruritic rash on the trunk as well as on the upper and lower extremities. Examination was notable for lichenified papules throughout the trunk and extremities, most notably on the back. Biopsies of the rash showed mild spongiosis with an underlying superficial and deep perivascular infiltrate (Fig 1). Due to failure of topical halobetasol, topical tacrolimus, oral antihistamines, prednisone, and mycophenolate mofetil, dupilumab was initiated at standard dosing. Within 3 months, the patient noticed a dramatic improvement of his rash and pruritus, complaining only of mild pruritus between injections and minimal residual post-inflammatory hyperpigmented macules. Dupilumab was stopped after 1 year due to insurance reasons, and the initial pruritic rash returned. After insurance reapproval, dupilumab was restarted with complete resolution of his rash and pruritus.

### Case 2

**Fig 2:**
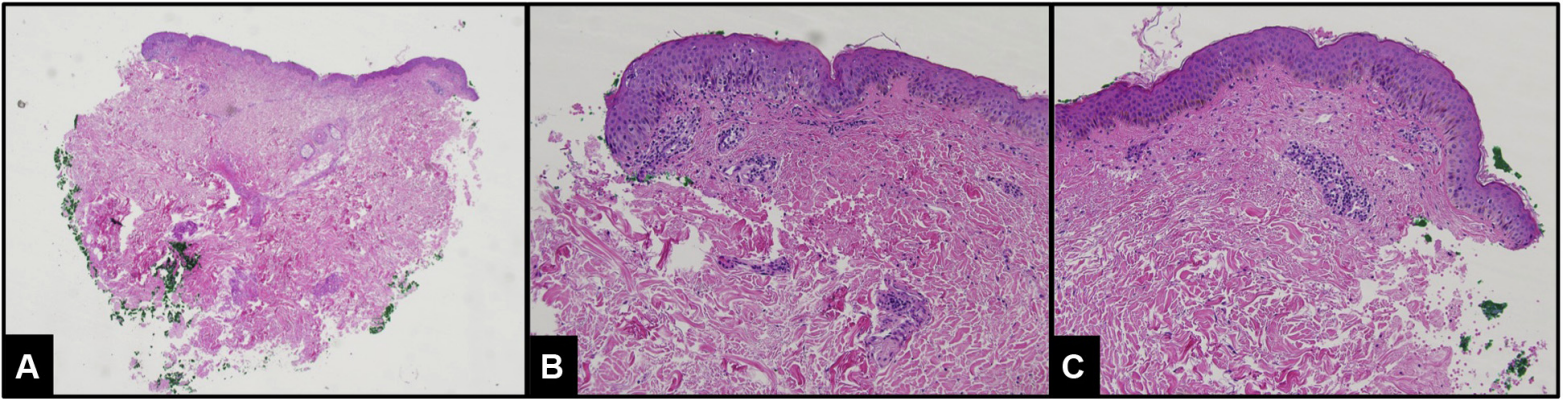
Punch biopsy of the right chest of patient case 2. Demonstrations of mild epidermal spongiosis with a scant perivascular lymphocytic infiltrate and rare dermal eosinphilis. (**A**, **B**, and **C**, Hematoxylin-eosin stain; original magnifications: **A** ×4; **B**, ×20; and **C**, ×20.)

A 48-year-old woman presented with a 5-year history of intense pruritus and rash significantly impacting her daily life. Examination showed few excoriated papules and subtle lichenification on the upper back, elbows, dorsal forearms, thighs, and fingers. Biopsy revealed mild epidermal spongiosis with a perivascular lymphocytic infiltrate containing rare eosinophils, consistent with DHR (Fig 2). After failing multiple therapies including topical betamethasone, topical tacrolimus, and oral mycophenolate mofetil, dupilumab was initiated with improvement in severity and duration of flares within the first 6 months. Due to slight progression of her baseline blurry vision and headaches, the dose was decreased to 200 mg every 2 weeks. The patient experienced subsequent flaring of her rash, so the dose was increased back to 300 mg every 2 weeks with resolution of her pruritus and rash and no further exacerbation of her ocular symptoms. Ultimately her ocular symptoms were evaluated by an ophthalmologist and deemed to not be consistent with dupilumab-induced conjunctivitis nor glaucoma.

### Case 3

A healthy 43-year-old woman presented with a 1-year history of a pruritic rash affecting her legs and abdomen. On exam, the patient was noted to have erythematous, blanchable papules coalescing into small plaques on her abdomen and distal part of the legs. Biopsy of the rash revealed an unremarkable epidermis and superficial perivascular lymphocytes with abundant interstitial eosinophils consistent with DHR. Patch testing was performed, which was 2+ for nickel sulfate and 1+ for p-tert-butylphenol formaldehyde resin, but the rash was persistent even with allergen avoidance. After failing multiple topical regimens, including triamcinolone and clobetasol, as well as oral prednisone, the patient was initiated on mycophenolate mofetil therapy, with excellent control but poor gastrointestinal tolerance. Her rash subsequently recurred, so dupilumab was started at standard dosing, and 5 months after starting dupilumab the patient's rash and pruritus had resolved without any side effects.

### Case 4

**Fig 3:**
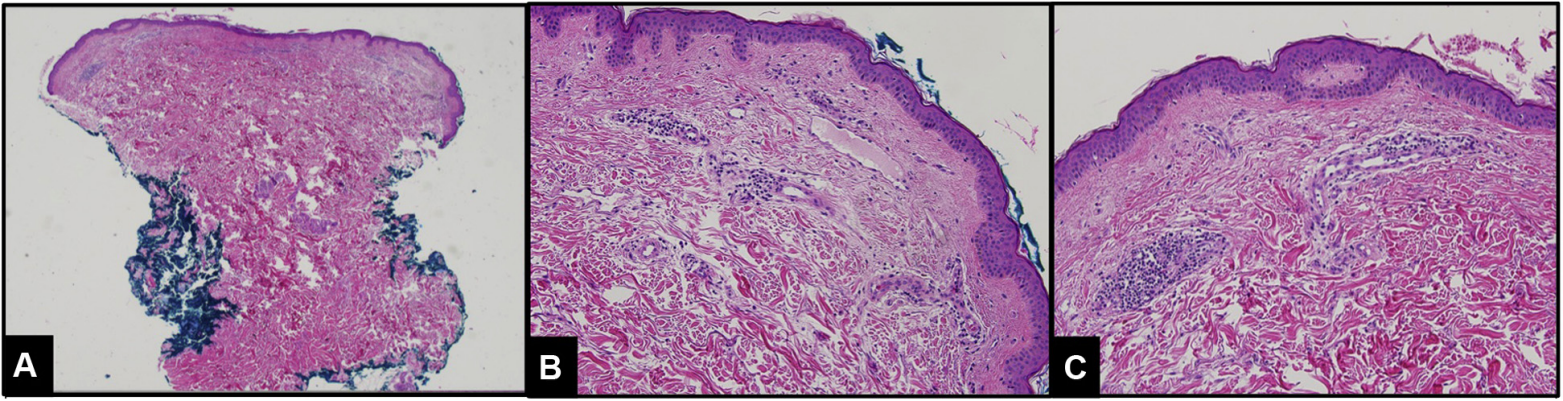
Punch biopsy of the right shoulder of patient case 4. Demonstrations of unremarkable epidermis and a dermis containing sparse perivascular lymphocytes as well as scattered interstitial eosinophils and neutrophils. (**A**, **B**, and **C**, Hematoxylin-eosin stain; original magnifications: **A**, ×2; **B** ×10; and **C**, ×10.)

A 68-year-old man presented with a 6-month history of a pruritic rash that began on his back and legs and spread to his knees, elbows, shoulders, and chest. Patch testing showed 1+ positivity for both sodium laurel sulfate and benzaprene #4, which were deemed not clinically relevant. On examination, he had scattered erythematous scaly patches on the upper chest, shoulders, and back with overlying excoriation. Biopsy of the right shoulder showed an unremarkable epidermis and a sparse perivascular and interstitial mixed infiltrate containing scattered interstitial eosinophils, consistent with a DHR (Fig 3). Oral prednisone initially cleared the rash, but it recurred on discontinuation. The rash was also recalcitrant to trials of topical steroids, oral antihistamines, and topical tacrolimus; therefore, he was transitioned to dupilumab at standard dosing. After 3 months, the patient reported complete clearing of the rash and pruritus. He did note occasional eye dryness, which was well-managed with artificial tears.

### Case 5

A 75-year-old man presented with a 1-year history of recurrent diffuse, pruritic rash. Examination revealed a generalized eruption of erythematous papules with minimal scale on the extremities and trunk particularly the flanks. Initial differential diagnosis included hypersensitivity dermatitis, contact dermatitis, non-bullous pemphigoid, atopic dermatitis, and Grover disease. A biopsy was performed on the left part of the chest and revealed a predominantly perivascular inflammatory infiltrate with occasional eosinophils consistent with DHR. A direct immunofluorescence test was negative. The patient failed multiple therapies, including topical triamcinolone, clobetasol, and hydroxyzine. Oral prednisone helped but was discontinued due to steroid-induced diabetes. The patient was started on dupilumab 300 mg injections every 14 days and within 4 months, his dermatitis and pruritus resolved. Due to cost, the injections were spaced to every 30 days, and he continued to experience resolution of his symptoms without any side effect from the medication.

### Case 6

A 78-year-old man presented with a 5-year history of extreme pruritus. No significant dermatitis was observed aside from faint pink patches on the upper chest and lower back with mild lichenification. Patch testing was performed and revealed 1+ positivity to potassium dichromate, but no culprit allergens were identified. A biopsy taken from the right part of the chest revealed mild acanthosis and minimal spongiosis with a perivascular lymphocytic infiltrate containing rare eosinophils in the presence of a negative direct immunofluorescence test, consistent with a DHR. The patient failed multiple therapies including topical corticosteroids, antihistamines, doxepin, narrow-band UV-B light, doxepin, gabapentin, butorphanol, and aprepitant. Azathioprine was poorly tolerated due to fatigue. The patient was then started on dupilumab with dramatic improvement in his pruritus and a 50%-60% reduction of the rash within the first 2 months with no associated side effects. His condition remains stable on this medication.

Additional clinical information is summarized in Table I.

**Table I:** Patient characteristics

Age at diagnosis	Gender	Comorbidities	Location of rash	Morphology of initial rash	Clinical differential diagnosis	Path report comments	Additional work up	Previous therapies	Outcomes	Side effects of dupilumab
53	M	Hypertension, dyslipidemia, hypereosinophilia, membranous nephropathy with severe refractory nephrotic syndrome	Trunk and extremities	Pink, lichenified papules	Dermal hypersensitivity reaction, arthropod bite reaction, resolving spongiotic dermatitis, atopic dermatitis (AD), IgG4 disease	Mildly spongiotic epidermis with lymphocytic inflammatory infiltrate with numerous eosinophils surrounding the vessels of the papillary dermis (Fig 1)	Periodic acid–Schiff negative. White blood count with slight neutrophilia. Creatinine 2.3, Blood urea nitrogen, 27.	Prednisone, mycophenolate mofetil, cetirizine, halobetasol, tacrolimus	Resolution of rash and pruritus	None
48	F	Ileal tumor, menorrhagia, acute deep venous thrombosis of left gonadal vein, anxiety, bipolar depression	Upper part of the back, elbows, dorsal forearms, thighs, and fingers (especially dorsal aspects of the metacarpophalangeal joints and proximal interphalangeal joints)	Excoriated papules with subtle lichenification	Hypersensitivity dermatitis, latex allergy, irritant dermatitis, contact dermatitis, infectious disease reaction, dermatomyositis	Mild epidermal spongiosis with a scant perivascular lymphocytic infiltrate and rare dermal eosinophils (Fig 2)	No histologic evidence of dermatomyositis. Complete blood count/Comprehensive metabolic panel within normal limits.	Mycophenolate mofetil, prednisone, tacrolimus, betamethasone, calamine lotion, Neosporin, coconut oil, bleach bath, mupirocin	Resolution of rash and pruritus	Blurry vision (determined to be chronic), headache
43	F	None	Abdomen and lower legs	Erythematous, blanchable papules coalescing into small plaques	Hypersensitivity dermatitis, granuloma annulare, mucin deposition disease, contact dermatitis, nummular eczema, AD, drug reaction, infectious process	Unremarkable epidermis and a dermis with superficial perivascular lymphocytes and abundant interstitial eosinophils	Patch testing was minimally positive for nickel sulfate (2+) and p-tert-butylphenol formaldehyde resin (1+). Complete blood count/Comprehensive metabolic panel/Thyroid-stimulating hormone within normal limits. Negative Hepatitis B surface antigen, quantiferon, and HIV antibody tests.	Prednisone, mycophenolate mofetil, hydroxyzine, triamcinolone, nystatin-hydrocortisone-zinc oxide, fluconazole, clobetasol, cetirizine, cephalexin	Resolution of rash and pruritus	None
68	M	Hypertension, hyperlipidemia, peripheral artery disease, coronary artery disease, gastroesophageal reflux disease, chronic obstructive pulmonary disease	Upper part of the chest, shoulder, back	Scattered erythematous scaly patches with overlying excoriation	AD, dermatitis herpetiformis, chronic pruritus secondary to other causes, dermal hypersensitivity reaction	Unremarkable epidermis and a dermis containing sparse perivascular lymphocytes as well as scattered interstitial eosinophils and neutrophils (Fig 3)	Patch testing showed minimal positivity for sodium laurel sulfate (1+) and benzaprene #4 (1+).	Tacrolimus, clobetasol, halobetasol, prednisone, chlorpheniramine	Resolution of rash and pruritus	Eye dryness
75	M	Coronary artery disease, steroid-induced diabetes mellitus, asthma, sarcoidosis	Trunk, particularly the flanks	Pink, red dermatitis with mostly broad patches of erythema and slight scale but some more papular components as well	Hypersensitivity dermatitis, contact dermatitis, non-bullous pemphigoid, mastocytosis, AD, Grover disease (with papular component)	Predominately perivascular inflammatory infiltrate with occasional dermal eosinophils	Grocott's Methenamine Silver stain negative. Direct immunofluorescence studies are negative.	Clobetasol, nystatin cream, triamcinolone cream, prednisone, hydroxyzine	Resolution of rash and pruritus	None
78	M	Coronary artery disease, gout, peripheral artery disease, cerebrovascular accident	Upper part of the chest, lower back	Faint pink patchy dermatitis with slight lichenification, minimal papular component, no scale	Dermatitis herpetiformis, bullous pemphigoid, hyper-eosinophilic syndrome	Mild acanthosis and spongiosis of the epidermis Superficial perivascular lymphocytes and rare interstitial eosinophils	Direct immunofluorescence studies negative. Patch testing minimal positivity to potassium dichromate (1+). Hemoglobin low at 12.9. Platelets/White blood count normal. Comprehensive metabolic panel normal. Negative Hepatitis B surface antigen and HIV antibody. Immunohistochemistry, including serum free kappa/lambda light chain, within normal limits.	Phototherapy, doxepin, mirtazapine, gabapentin, butorphanol, hydroxyzine, aprepitant, clobetasol, tacrolimus, oral prednisone, mycophenolate mofetil, azathioprine	Occasional pruritus, 50%-60% improvement of rash on physical exam	None

*AD*, Atopic dermatitis; *F*, female; *IgG*, immunoglobulin; *M*, male

## Discussion

Though DHR presents variably, the most common primary lesions associated with DHR are often papules, papulovesicles, plaques, patches, or erythema.^1^ Similarly, CPUO can present with small, pink “micropapules” in a generalized distribution.^2^ All 6 of the cases presented here had a papular component to the dermatitis as well as pruritus and erythema. The location of the rashes varied by case, but spared the face, palms, and soles in all cases.

Extensive work up to exclude alternative underlying etiologies was performed in all cases. Laboratory work up was unrevealing, although 2 of 6 patients showed peripheral eosinophilia. Patch testing was performed in 3 cases, notable for 2+ nickel sulfate and 1+ p-tert-Butylphenol formaldehyde resin; 1+ sodium laurel sulfate and benzaprene #4; and 1+ potassium dichromate, respectively. Allergens were deemed to not be clinically relevant, however.

All 6 cases included in this study had evidence of DHR on biopsy with a perivascular inflammatory infiltrate with eosinophils, which was an inclusion criterion for this study. Three of the 6 cases had mild focal spongiosis noted in the pathology report as well, though not to a degree observed in eczematous dermatitis. Secondary change of acanthosis and lichenification was noted in 1 of the 6 cases.

Side effects have been reported with dupilumab therapy, including injection site reaction, conjunctivitis, and upper respiratory tract infections.^4^ In this case series, 1 patient experienced dry eyes, and another patient experienced blurry vision, though the blurry vision was ultimately determined to be chronic rather than a side effect of the medication.

Though previously refractory to multiple oral and topical therapies, all 6 patients in this study experienced significant improvement of their symptoms while using dupilumab, and 5 experienced complete resolution of their rash and pruritus. Outside of this case series, there is only 1 published case report documenting the successful use of dupilumab in a patient with chronic pruritus and DHR on biopsy.^5^ Based on these 6 cases and the prior report,^5^ dupilumab may be a new treatment approach for patients with treatment refractory CPUO with DHR on biopsy.

## Conflicts of interest

None disclosed.
